# Tumor vascular disruption using various radiation types

**DOI:** 10.7717/peerj.320

**Published:** 2014-04-01

**Authors:** JJ Bevelacqua

**Affiliations:** Bevelacqua Resources, USA

**Keywords:** Absorbed dose, Dose assessment, Radiation therapy, Tumor vascular disruption, Microspheres

## Abstract

The feasibility of disrupting a tumor’s vascular structure with various radiation types and radionuclides is investigated. Calculated absorbed dose profiles for photons and ^4^He ions suggest that low-energy beta-gamma and alpha emitting radionuclides can deposit sufficient absorbed dose to disrupt a tumor’s vascular structure while minimizing the dose outside the blood vessel. Candidate radionuclides uniformly distributed in microspheres are theoretically investigated with respect to their vascular disruption potential and to offer an alternative to ^90^Y microsphere therapy. Requisite activities of candidate low-energy beta-gamma and alpha emitting radionuclides to facilitate vascular disruption are calculated.

## Introduction

Pilat & LoRusso (2006) note that current therapy methodologies for the treatment of most solid tumors are resulting in diminishing returns. Although their focus is on chemotherapy, the arguments are also applicable to radiotherapy approaches. For example, a symptomatic solid tumor usually contains 10^9^–10^11^ cells. These cells must be destroyed or the treatment only results in a temporary tumor growth delay.

Another issue with existing therapy approaches is that agents that deliver dose to tumor cells also irradiate healthy tissue. The irradiation of healthy tissue following the administration of therapeutic administrations of radionuclides affects the patient’s quality of life and leads to short-term as well as long-term detriments. Although the short-term detriment varies with the specific therapy approach, it is illustrated with radiotherapy methods for prostate cancer. The short-term effects include incontinence and erectile dysfunction that affects the patient’s recovery and subsequent quality of life (Martinez et al., 2000). Long-term effects of radiotherapy can include secondary cancers and cardiovascular disease (NCRP, 2012). For these reasons, it is important to continue to investigate alternative radiotherapy approaches that deliver dose selectively to the target tissue (i.e., tumor mass or vascular structure) and minimize dose to healthy tissue.

One approach that has the potential to impact tumor cells involves disrupting its vascular structure. A number of authors (Folkman, 1971; Burke & DeNardo, 2001) have proposed the basis for a therapy approach that prevents the development of the tumor’s vascular supply. Vascular disruption agents incorporate both chemotherapy (Carmeliet & Jain, 2000; Rajput & Agrawal, 2010) as well as radiotherapy (Kennedy & Salem, 2003; Carr, 2004; Murthy et al., 2005; Kennedy et al., 2007; Chaudhury et al., 2010; Rajput & Agrawal, 2010; Dezarn et al., 2011; Lau, Lai & Leung, 2011; Lee & Seong, 2012; Amor-Coarasa et al., 2014). These approaches are also known as anti-angiogenic or radioembolization therapies.

Radiotherapy vascular disruption techniques have been extensively applied to liver cancers (Kennedy & Salem, 2003; Carr, 2004; Murthy et al., 2005; Kennedy et al., 2007; Chaudhury et al., 2010; Rajput & Agrawal, 2010) utilizing ^90^Y microspheres. Other radionuclides (e.g., ^32^P) have been less thoroughly investigated and radiation types other than high-energy beta particles have not been systematically investigated (Rajput & Agrawal, 2010).

This paper investigates other radiation types and radionuclides that offer additional flexibility in disrupting a tumor’s vascular structure. These radionuclides should selectively deliver absorbed dose to the tumor’s vasculature. Unlike the ^90^Y approach that has been successfully applied in a clinical setting, the proposed approach is theoretical and has yet to be utilized in treatment applications.

## Tumor Vasculature

One of the most striking characteristics of solid tumors is their vascular configuration (Carmeliet & Jain, 2000). In normal tissues, the vasculature structure is arranged to provide optimum nourishment conditions. In general, growing tumors have a chaotic vasculature that is not fully developed or adequate to optimally nourish the tumor cells. Given this condition, the tumor’s vasculature can be disrupted with an appropriate agent.

Siemann (2002) notes that common defects in a tumor’s vascular structure include vessels that are dilated and have elongated shapes, blind ends, bulges, leaky sprouts, and abrupt changes in diameters. Accordingly, blood flow in these vessels is sluggish and irregular. This flow pattern provides less nourishment than delivered to normal cells and results in hypoxic areas that are characteristic of solid tumors (Siemann, 2002). These hypoxic conditions limit the effectiveness of both chemotherapy and radiotherapy (Siemann, 2002; Wouters et al., 2002). The lack of oxygen provides a degree of radioresistance to tumor cells when compared with oxygenated tumor cells. Since a tumor’s growth is dependent on sufficient nourishment, its viability is affected by disrupting the blood supply. In principle, eliminating a tumor’s blood supply provides an alternative therapy approach to facilitate or supplement its destruction.

## Current Radiological Efforts

Radiological efforts at tumor vascular disruption have focused on ^90^Y . ^90^Y was a logical choice for anti-angiogenic therapy since the dose (≥70 Gy) for the vascular disruption of a tumor (Kennedy et al., 2007) is easily achieved using ^90^Y .

An anti-angiogenic approach to heptocellular carcinoma has successfully incorporated ^90^Y . However, the ^90^Y beta particles have significant range and extend well beyond the vascular target. The maximum ^90^Y beta energy of 2.27 MeV has a range in tissue of about 1.1 cm, which delivers absorbed dose beyond the target vascular structure. Additional dose is delivered by the associated ^90^Y bremsstrahlung radiation.

Commercially available products utilize microspheres incorporating ^90^Y to preferentially lodge in the tumor’s vasculature. For example, resin microspheres (Bilbao & Reiser, 2008) are a permanent implant containing ^90^Y . Glass microspheres have also been utilized in therapy applications (Kennedy & Salem, 2003; Carr, 2004). The properties of currently available microspheres are summarized in Table 1.

**Table 1:** Properties of resin and glass ^90^Y microspheres.

Parameter	Microsphere type	
	Resin	Glass
Diameter (µm)	20–60	20–30
Density (g/cm^3^)	1.6	3.6
Activity per microsphere (Bq)	50	2500
Number of microspheres per 3 GBq vial (×10^6^)	40–80	1.2
^90^Y form	Yttrium bound to resin	Yttrium in glass matrix

**Notes.**

Derived from Kennedy et al. (2007).

## Methods

Alternatives to ^90^Y microspheres are investigated by examining various radiation types and radionuclides to deliver absorbed dose preferentially to a tumor’s vasculature. This is accomplished in a two-fold manner. The first method investigates various radiation types using a conceptual treatment approach that utilizes a postulated internal radiation-generating device based on advanced nanotechnology. A second method uses the results of the internal radiation-generating device calculations to suggest radionuclides that could be incorporated into microspheres to disrupt a tumor’s vasculature. In comparison to the ^90^Y approach, these radionuclides would selectively deliver absorbed dose to vascular tissue and reduce the dose delivered to healthy tissue.

The first method utilizing radiation-generating devices (Bevelacqua, 2005; Bevelacqua, 2010a; Bevelacqua, 2012) involves technology that does not yet exist. However, this technology is moving closer to fruition. Travish & Yoder (2011) have developed laser-driven electron accelerators about the size of the eye of a needle. These accelerators use optical cavities that will optimally have a size on the order of the radiation’s wavelength. Using shorter wavelength radiation would bring the scale of these devices to the size envisioned for internal radiation-generating devices.

The internal radiation-generating device treatment concept has the potential to preferentially deliver absorbed dose to the tumor vasculature, but requires significant development. As such, it is a theoretical approach that will become more viable as technological advances occur. However, internal radiation-generating device calculations suggest a near-term alternative to ^90^Y microspheres.

Alternative microspheres incorporating nuclides that emit radiation types with a shorter range than ^90^Y are evaluated. These shorter-range radiation types deliver less dose to tissue beyond the target vascular tissue.

### Experimental approach

The proposed microsphere approach is currently limited to a theoretical treatment. This is expected since this initial investigation determines the appropriate radionuclides and radiation types and associated energies to ascertain the viability of the technique. The present work determines these parameters, which is the first step in developing the proposed microsphere vascular disruption therapy approach. The next step is the detailed design of the microsphere in terms of the specific materials of construction, optimum size, and radionuclide distribution within the microsphere to determine the deposition characteristics within the target blood vessel. Experimental work would follow the completion of the aforementioned tasks and construction of the designed microspheres loaded with candidate radioactive material.

These theoretical steps are necessary to develop the requisite parameters to determine a viable experimental approach. Accordingly, no experimental results are provided in this paper.

This situation is similar to the development of an antiproton therapy modality (Holzscheiter et al., 2006; Bittner et al., 2013). Theoretical calculations were the first step in establishing the feasibility of the antiproton therapy method. These calculations required a number of years to refine and establish the appropriate parameters for the design of an experimental approach.

### Theoretical approach

Disrupting a tumor’s vascular structure using radiotherapy could be performed as a stand-alone protocol, as the first step in a protocol followed by a chemical vascular disrupting agent (VDA), as a final step in a therapy procedure initiated by a VDA, or as part of an alternating sequence involving both radiotherapy and VDAs. This paper does not select a specific therapy approach, and only investigates candidate radionuclides and radiation types that could be used to disrupt a tumor’s vasculature. As such, the present work is strictly theoretical, but offers the potential for enhancing existing tumor treatment approaches.

The blood supply to a tumor could be disrupted by damaging the vessel wall, causing the vessel to become restricted, or increasing its leakage. Table 2 summarizes the geometry of a variety of human blood vessel types (Barrett et al., 2012) including those that could service a developing tumor. A review of the literature suggests developing tumor vessel wall sizes will typically be less than 100 µm (Less et al., 1991; Barrett et al., 2012). This wall size includes arterioles (see Table 2), which are the assumed base case for the calculations presented in this paper.

**Table 2:** Characteristics of various blood vessel types.

Blood vessel type	Wall thickness	Lumen diameter
Aorta	2 mm	25 mm
Artery	1 mm	4 mm
Arteriole	20 µm	30 µm
Capillary	1 µm	8 µm
Venule	2 µm	20 µm
Vein	0.5 mm	5 mm
Vena Cava	1.5 mm	30 mm

**Notes.**

Derived from Barrett et al. (2012).

### Absorbed dose computational model

Internal radiation-generating devices are a potential vehicle to deliver various radiation types to the tumor vasculature. The theoretical design characteristics and capabilities of internal radiation-generating devices (Bevelacqua, 2005; Bevelacqua, 2010a; Bevelacqua, 2012) are used to investigate the vascular disruption characteristics of protons, ions, and low-energy photons.

### Protons and ions

For a tissue volume irradiated by a beam of ions of a given energy, the absorbed dose (*D*) as a function of penetration distance *x* into tissue is (Kraft, 2000; Bevelacqua, 2005):

(1) \begin{eqnarray*} \displaystyle D(x)=\frac{1}{\rho }\left(-\frac{d E}{d x}\right)\Phi (x)&&\displaystyle \end{eqnarray*}

where *ρ* is the density of the material (tissue, tumor, or other structure) attenuating the ion, −*dE*/*dx* is the stopping power, and Φ(*x*) is the ion fluence. The particle fluence varies with tissue penetration depth according to the relationship:

(2) \begin{eqnarray*} \displaystyle \Phi (x)=\Phi (0){e}^{-\Sigma x}&&\displaystyle \end{eqnarray*}

where Φ(0) is the entrance fluence and Σ is the macroscopic reaction cross-section.

Ion stopping powers are determined using Bethe’s formulation (Bethe, 1930) and follow an approach similar to SPAR (RSICC, 1985). The energy dependent cross-sections are obtained from Shen’s parameterization (Shen et al., 1989).

Calculations are performed for photon, proton, ^4^He, ^12^C, ^20^Ne, and ^40^Ca beams delivered by internal radiation-generating devices (Bevelacqua, 2005; Bevelacqua, 2010a; Bevelacqua, 2012). All beams are assumed to be fully ionized (e.g., ^40^Ca ions have a +20 e charge).

### Low-energy photons

The photon absorbed dose is derived from the standard point source relationships (Bevelacqua, 2010b),

(3) \begin{eqnarray*} \displaystyle D=\frac{S}{4 \pi {r}^{2}}\hspace{0.167em} \frac{{\mu }_{\mathrm{en}}}{\rho }E B(\mu x){e}^{-\mu x}&&\displaystyle \end{eqnarray*}

where *S* is the total number of photons that irradiates the arteriole wall, *r* is the distance from the radiation-generating device, *μ*_en_/*ρ* is the mass-energy absorption coefficient, *E* is the photon energy, *B* is a buildup factor, and *μ* is the attenuation coefficient. Low-energy photon beams are also evaluated using the mass energy absorption coefficients and buildup factors derived from Grodstein (1957) and Trubey (1988). Since higher-energy photons have poor spatial localization, low-energy photons are evaluated as a possible vascular disruption agent.

## Results and Discussion

Since the base case considered in this paper is the 20 µm thickness of an arteriole wall, the focus is delivering a requisite dose to this tissue region and for wall thicknesses ≤100 µm that service most tumors (Less et al., 1991; Barrett et al., 2012). The target dose delivered to this tissue is assumed to be sufficient to disrupt the vessel wall, which is on the order of 100 Gy (Kennedy et al., 2007). No attempt to optimize dose delivery has been made. Ion fluences of 5 × 10^9^, 5 × 10^8^, 1 × 10^8^, 5 × 10^7^, and 1 × 10^7^ ions/cm^2^ for protons, alpha particles, ^12^C, ^20^Ne, and ^40^Ca, respectively and 1 × 10^10^ photons are utilized in the subsequent calculations.

**Figure 1:**
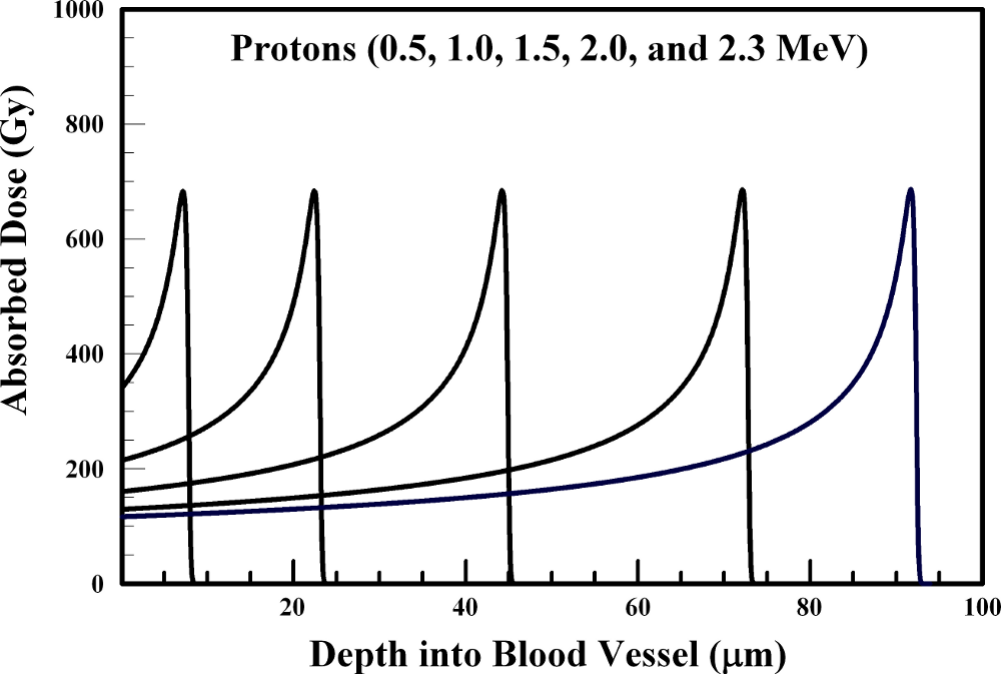
Absorbed dose profiles for 0.5 (far left curve), 1.0, 1.5, 2.0, and 2.3 (far right curve) MeV protons in water. The absorbed dose curves peak at a greater depth with increasing proton energy. The total proton fluence for all energies is 5.0 × 10^9^ p/cm^2^. The protons are delivered by an internal radiation-generating device.

**Figure 2:**
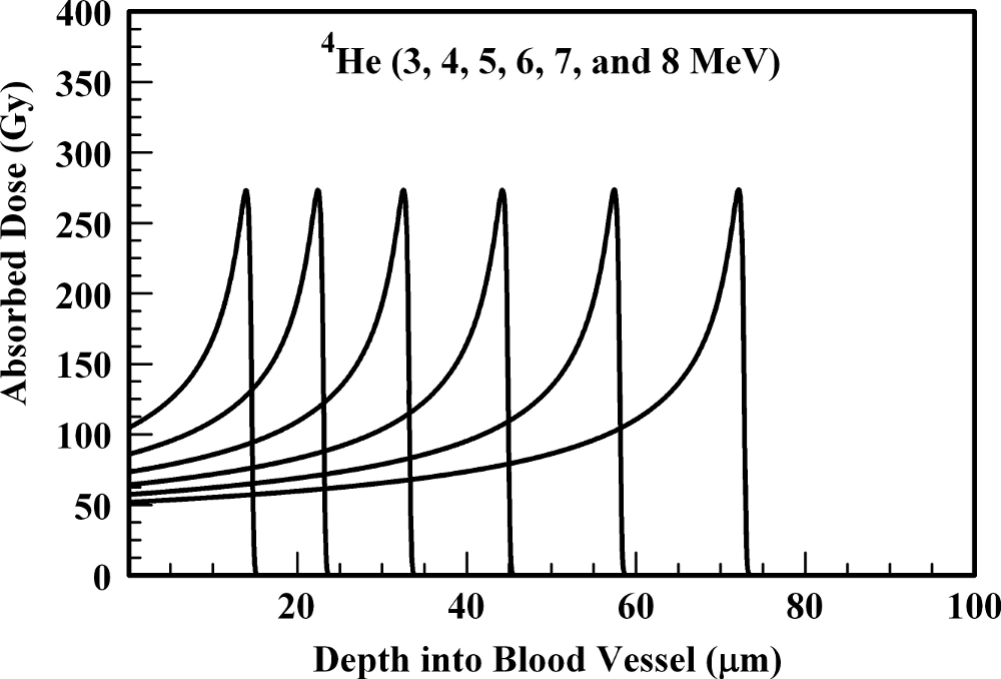
Absorbed dose profiles for 3.0 (far left curve), 4.0, 5.0, 6.0, 7.0, and 8.0 (far right curve) MeV ^4^He ions in water. The absorbed dose curves peak at a greater depth with increasing ^4^He ion energy. The total ion fluence for all energies is 5.0 × 10^8^ ^4^He ions/cm^2^. The ions are delivered by an internal radiation-generating device.

**Figure 3:**
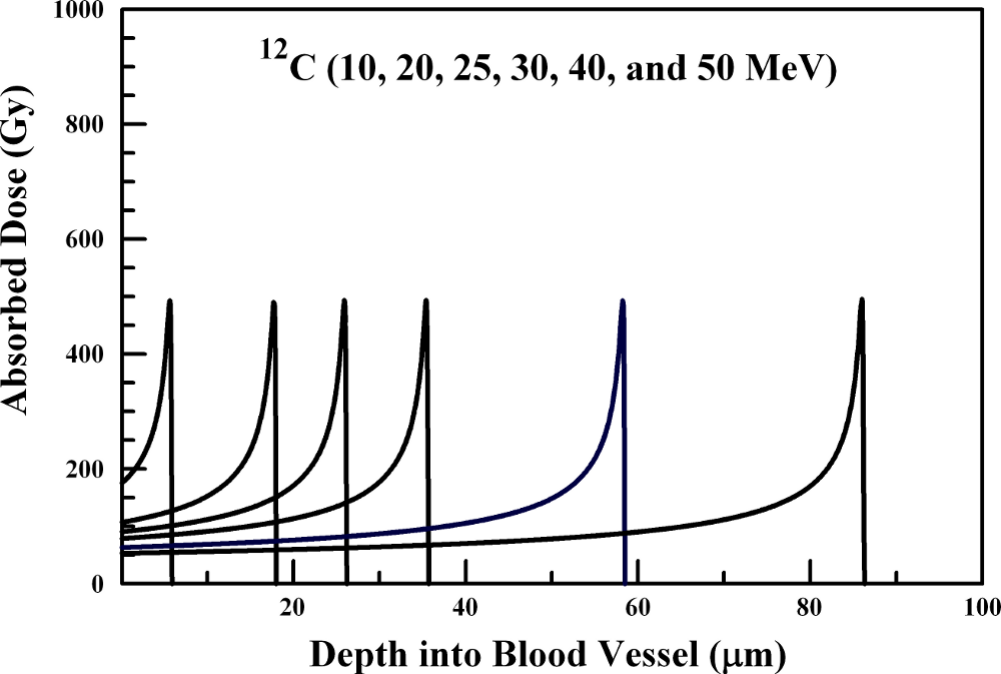
Absorbed dose profiles for 10.0 (far left curve), 20.0, 25.0, 30.0, 40.0, and 50.0 (far right curve) MeV ^12^C ions in water. The absorbed dose curves peak at a greater depth with increasing ^12^C ion energy. The total ion fluence for all energies is 1.0 × 10^8^ ^12^C ions/cm^2^. The ions are delivered by an internal radiation-generating device.

**Figure 4:**
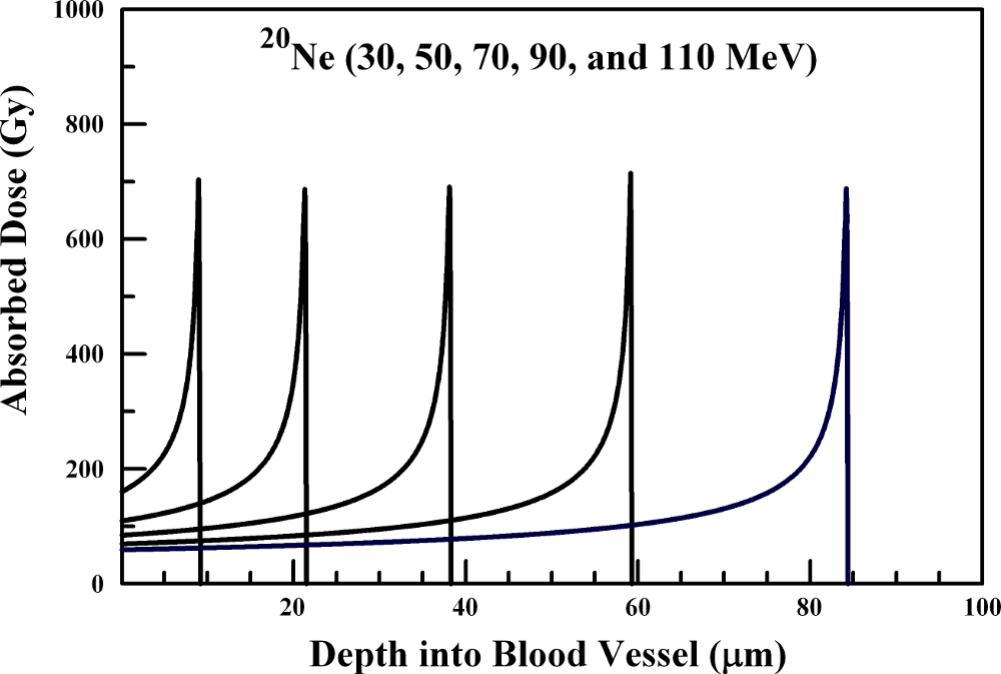
Absorbed dose profiles for 30.0 (far left curve), 50.0, 70.0, 90.0, and 110.0 (far right curve) MeV ^20^Ne ions in water. The absorbed dose curves peak at a greater depth with increasing ^20^Ne ion energy. The total ion fluence for all energies is 5.0 × 10^7^ ^20^Ne ions/cm^2^. The ions are delivered by an internal radiation-generating device.

**Figure 5:**
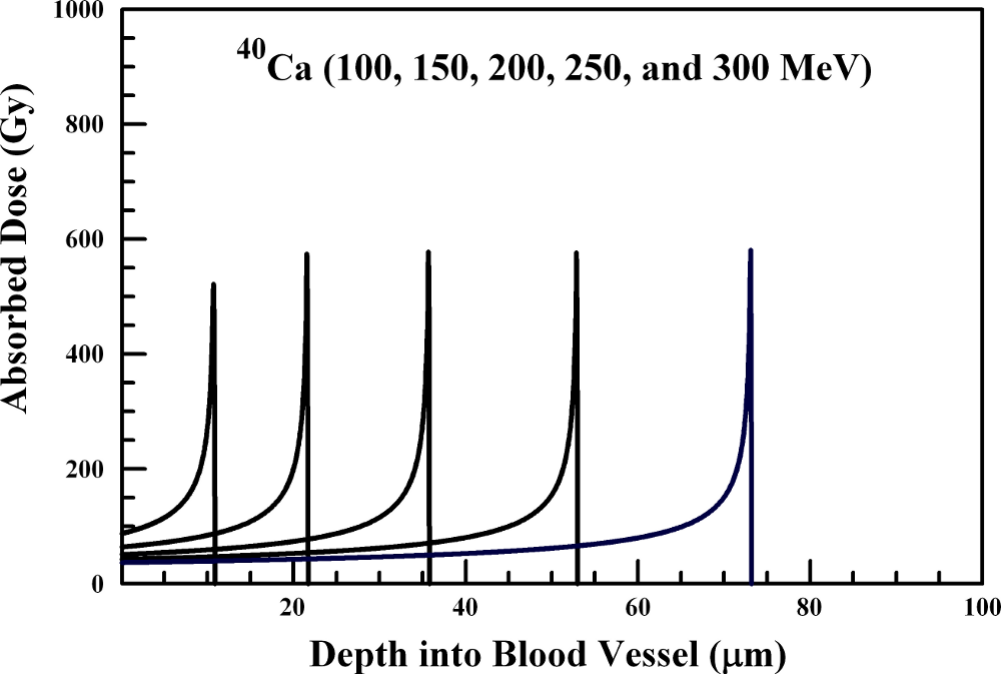
Absorbed dose profiles for 100.0 (far left curve), 150.0, 200.0, 250.0, and 300.0 (far right curve) MeV ^40^Ca ions in water. The absorbed dose curves peak at a greater depth with increasing ^40^Ca ion energy. The total ion fluence for all energies is 1.0 × 10^7^ ^40^Ca ions/cm^2^. The ions are delivered by an internal radiation-generating device.

**Figure 6:**
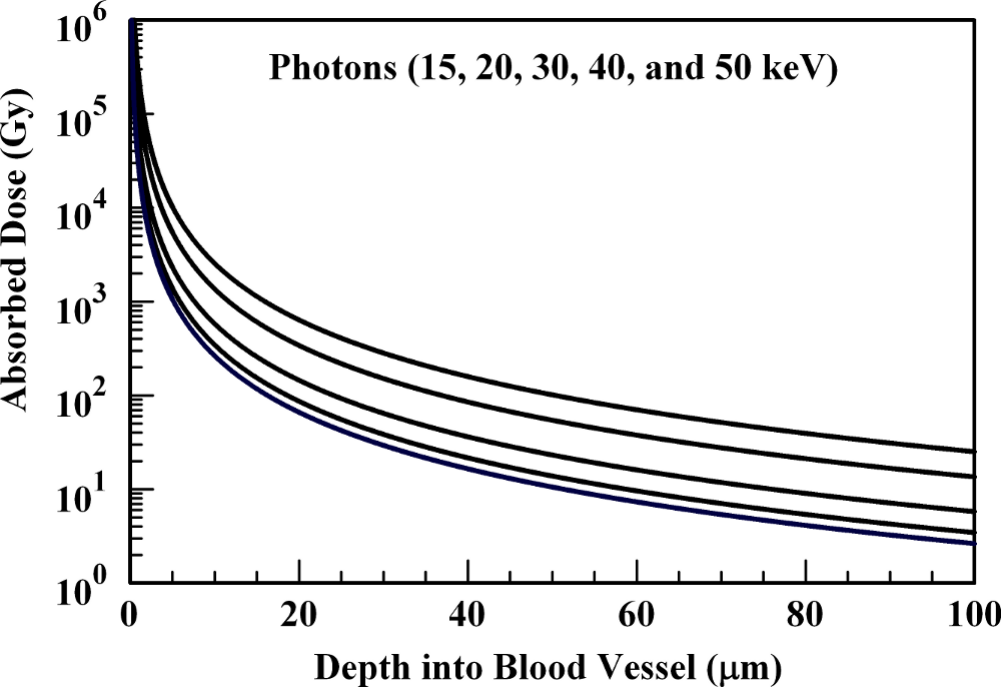
Absorbed dose profiles for 15.0 (top curve), 20.0, 30.0, 40.0, and 50.0 (bottom curve) keV photons in water. The absorbed dose curves decrease in magnitude with increasing photon energy. 1.0 × 10^10^γ are delivered by the internal radiation-generating device.

In performing the absorbed dose calculations, the internal radiation-generating device is assumed to reside at the inner blood vessel wall. The results of Figs. 1–6 provide absorbed dose profiles for blood vessel wall thickness values ≤100 µm. Water is assumed to be the medium comprising the vessel wall which is a reasonable tissue surrogate for initial calculations (Kraft, 2000; Bevelacqua, 2005).

### Protons

Figure 1 provides absorbed dose profiles for protons with energies between 0.5 and 2.3 MeV. Dose localization within an arteriole wall could be achieved using a 1.0–1.5 MeV proton beams. The results of Fig. 1 suggest the 100 Gy target dose for blood vessel destruction can be achieved using low-energy protons. These results also suggest that delivering the absorbed dose to the 20–100 µm depth is achieved using protons with energies between 1.0 and 2.3 MeV.

### ^4^He ions

Figure 2 summarizes ^4^He absorbed dose curves for 3–8 MeV alpha particles. These energies correspond to the values achieved by alpha particles emitted by many radionuclides. These results suggest that sufficient absorbed dose at the requisite depths can be delivered by alpha energies below 8 MeV.

Alpha particles with energies below 3 MeV will not penetrate the arteriole wall. The arteriole wall can be disrupted with minimal dose to surrounding tissue for alpha particles in the 4–5 MeV energy range. This energy range is obtained by numerous alpha-emitting radionuclides.

The results of Fig. 2 suggest that alpha emitting radionuclides could produce an alternative to the use of ^90^Y in microspheres. This alternative could be implemented in the near-term and would not require the advanced technology utilized in an internal radiation-generating device. The use of an alpha-emitting radionuclide in a microsphere to affect tumor vascular disruption is addressed in subsequent discussion.

### ^12^C, ^20^Ne, and ^40^Ca ions

Ion beams of ^12^C, ^20^Ne, and ^40^Caions and their penetration through the 20–100 µm range are summarized in Figs. 3, 4, and 5, respectively. ^12^C ions below about 20 MeV will not penetrate the arteriole wall, and 20–50 MeV ions will deposit sufficient energy into a range of vessel wall thicknesses in the 20–100 µm range to produce vascular disruption. Arteriole wall disruption can be achieved using 20–30 MeV ^12^C ions. However, generation of ^12^C, ^20^Ne, and ^40^Ca ions would present a greater challenge that producing lighter ions in a first generation internal radiation-generating device.

^20^Ne ions below 30 MeV will not penetrate the arteriole wall. As illustrated in Fig. 4, ^20^Ne ions in the range of 50–110 MeV will be sufficient to reach the range of vessel wall thicknesses addressed in this study. Arteriole wall disruption is achieved using 50–70 MeV ^20^Ne ions.

In a similar manner, ^40^Ca ions require of 150–200 MeV to disrupt the arteriole wall. Figure 5 illustrates the penetration of 100–300 MeV ^40^Ca ions through vessel wall thicknesses below 100 µm.

### Low-energy photons

Figure 6 illustrates that photon energies in the range of 15–50 keV can deposit the requisite absorbed dose to disrupt an arteriole wall. However, absorbed dose is also deposited in the 20–100 µm range by the 15–50 keV photons summarized in Fig. 6.

The results of Fig. 6 suggest that low energy gamma emitting radionuclides are also candidates to replace ^90^Y embedded in microspheres. These low energy gamma-emitting radionuclides will disrupt the tumor vascular structure and offer an alternative to the use of ^90^Y in microspheres. This possibility is addressed in subsequent discussion.

### Near-term therapy approach

The results of Figs. 1–6 suggest that the 100 Gy target dose to the arteriole wall can be achieved with the theoretical internal radiation-generating device utilizing a variety of radiation types and energies. This is an interesting result, but does not provide a viable, near-term therapy approach. However, the photon and alpha particle results offer the possibility of extending the ^90^Y microsphere approach to additional radionuclides that emit low-energy photons, low-energy beta particles, or alpha particles. The addition of low-energy beta particles to the candidate list is based on their expected range in tissue. These results are sufficient to suggest possible radionuclides that could be applicable as vascular disruption agents. These radionuclides would provide additional options for the radioactive material loading of microspheres used to disrupt a tumor’s vasculature.

In the subsequent discussion, a single microsphere is assumed to deliver the requisite disruption dose to the arteriole wall. Following the existing ^90^Y therapy approach, the total microsphere activity would be spread over a large number of microspheres (see Table 1) that are collectively designed to disrupt the tumor’s vasculature.

### Candidate microspheres

The internal radiation-generating device results of Figs. 2 and 6 offer insight to possible radionuclides that can be incorporated into microspheres to disrupt a tumor’s vasculature. These candidate microspheres are assumed to be loaded with either alpha or low-energy beta-gamma emitting radionuclides. Desirable characteristics for the radionuclide and candidate microsphere to facilitate tumor blood vessel disruption include:

1. The nuclide should have a short half-life. In this paper, an arbitrary half-life limit of 100 d is chosen.
2. The range of the radiation types emitted by the nuclide should be shorter than the range of the ^90^Y beta particles and their associated bremsstrahlung in tissue.
3. The absorbed dose delivered to the arteriole wall should be at least 100 Gy.
4. The dose delivered to healthy tissue should be minimized.
5. The microsphere has the capability to preferentially attach to the wall of an arteriole supplying blood to a tumor.
6. The candidate radionuclide is chemically compatible with a polymer microsphere that can be absorbed into the body. Absorption is assumed to occur after nearly all of the radioactive material decays.

Although these characteristics provide a basis for the calculations presented in this paper, they have not been optimized to produce a viable alternative to the ^90^Y microsphere approach. However, these characteristics provide an initial set of reasonable parameters to determine the properties of a replacement microsphere.

### Microspheres using alpha-emitting radionuclides

The properties of microspheres loaded with alpha-emitting radionuclides can be determined if a few simplifying assumptions are made. First, the microsphere is assumed to be small such that it provides minimal attenuation of the emitted alpha particles. Second, the alpha microsphere is assumed to attach preferentially and remain attached to the arteriole wall. Given these limitations, the dose rate delivered by the microsphere as a function of time *t*, $\dot {D}(t)$ is

(4) \begin{eqnarray*} \displaystyle \dot {D}(t)=\dot {D}(0){e}^{-\lambda t}&&\displaystyle \end{eqnarray*}

where *λ* is the physical disintegration constant for the alpha-emitting radionuclide, and *t* = 0 corresponds to the time that the microsphere attaches to the arteriole wall. Assuming the microsphere remains attached to the arteriole wall until all the radioactive material decays, the total dose (*D_T_*) delivered to the tumor vasculature is

(5) \begin{eqnarray*} \displaystyle {D}_{T}=\int \nolimits \nolimits _{0}^{\infty }\dot {D}(0){e}^{-\lambda t}d t=\frac{\dot {D}(0)}{\lambda }.&&\displaystyle \end{eqnarray*}

The initial dose rate can also be written in terms of a dose conversion factor *K* (ICRP, 2010)

(6) \begin{eqnarray*} \displaystyle \dot {D}\left(0\right)=\frac{A(0)}{4 \pi {r}^{2}}K&&\displaystyle \end{eqnarray*}

where *r* is the distance to the point of interest in the arteriole wall. Substituting the value of the initial dose rate from Eq. (5) into Eq. (6) yields the initial attached microsphere activity:

(7) \begin{eqnarray*} \displaystyle A(0)=\frac{4 \pi {r}^{2}\lambda {D}_{T}}{K}&&\displaystyle \end{eqnarray*}

where *D_T_* is the total dose delivered to the arteriole wall to facilitate vascular disruption (100 Gy), *r* is chosen to be 15 µm as a representative depth into the arteriole wall, and *K* is the ICRP 116 dose conversion coefficient (ICRP, 2010) corresponding to the emitted alpha particle energy. If the radionuclide emits multiple alpha particles, the dose conversion factor for the average alpha energy is used.

Table 3 provides the activities of candidate alpha microspheres that deliver 100 Gy at a depth of 15 µm into an arteriole wall supplying a tumor. The alpha particle energies, yields, and half-lives used in Table 3 are obtained from Shleien, Slaback & Birky (1998) and Baum et al. (2010). Only the parent nuclide decays are used in these initial calculations and no daughter contributions are considered.

**Table 3:** Candidate alpha emitting nuclides for loading microspheres.

Nuclide	Half-life	Activity to deliver 100 Gy at 15 µm depth in an arteriole wall (kBq)
^149^Tb	4.12 h^a^	0.152
^206^Pb	8.80 d^b^	0.00226
^211^At	7.214 h^b^	0.0588
^212^Bi	1.009 h^b^	0.407
^222^Rn	3.823 d^b^	0.00494
^223^Ra	11.43 d^b^	0.0016
^224^Ra	3.66 d^b^	0.00499
^225^Ac	10.00 d^b^	0.00179
^227^Th	18.718 d^b^	0.00094
^230^U	20.8 d^b^	0.00085
^240^Cm^c^	27.0 d^b^	0.00061
^246^Cf^c^	35.7 h^b^	0.0103
^253^Es^c^	20.47 d^b^	0.00076

**Notes.**

a Baum et al. (2010).

b Shleien, Slaback & Birky (1998).

c These nuclides are difficult to produce and would not represent likely microsphere candidates.

The radionuclides listed in Table 3 are somewhat arbitrary since they were limited to alpha energies in the 3–8 MeV range. The requisite alpha activities to accomplish arteriole disruption are in general smaller than the 0.05–2.5 kBq values used in the ^90^Y microspheres. This is expected since the calculated activities are values corresponding to their attachment on the arteriole inner wall. The manufactured activity will depend on the nature of the fabrication process, the time between fabrication and injection, and the time to attach to the arteriole wall following injection. However, the requisite activity levels appear to be achievable with current technology and suggest that alpha particle microspheres can be designed to implement tumor vascular disruption.

The calculations summarized in Table 3 are based on a first order approximation intended to provide an order of magnitude estimate of the requisite activity to initiate vascular disruption. A more sophisticated design effort is required to develop a viable alpha radionuclide microsphere therapy approach, but the results summarized in Table 3 suggest the alpha microsphere approach is viable in the near term and likely to be achieved before the development of equivalent internal radiation-generating devices.

One of the challenges of using alpha emitting radionuclides is their difficulty of production and associated availability. Therefore, the radionuclide selected to load the alpha microsphere should be readily available. As such, ^222^Rn is an attractive possibility if a microsphere can be designed to retain the gas. Gas retention is certainly achievable as evidenced by the retention of fission gasses in the ceramic uranium dioxide fuel pellet used in commercial power reactors (Bevelacqua, 2010b). The ^222^Rn daughters will yield additional dose to the tumor vasculature, which also enhances the approach. However, the calculations summarized in Table 3 only include radiation types emitted by the parent nuclide.

Some of the nuclides presented in Table 3 (e.g., ^240^Cm, ^246^Cf, and ^253^Es) are difficult to produce and would not represent likely microsphere candidates. These nuclides are presented to illustrate the impact of higher energy alpha particles in the range of 6–7 MeV.

The selected alpha-emitting radionuclide will have superior dose localization capability in a blood vessel wall when compared to ^90^Y . Designing an appropriate microsphere is dependent on the characteristics of the selected radionuclide and microsphere material and their physical and chemical characteristics.

### Microspheres using low-energy beta-gamma emitting radionuclides

The beta-gamma microsphere concept was investigated using the ISO-PC computer code (Rittmann, 2004). In modeling this option, the beta-gamma source activity was uniformly embedded in a 15 µmradius carbon microsphere with a density of 2 g/cm^3^. The model density and size of the microsphere lies within the range of the ^90^Y microspheres defined in Table 1. ISO-PC defined tissue is selected as the composition of the blood vessel material.

Given the small size of the microsphere and target arteriole wall, calculations are relatively insensitive to the model geometry (i.e., the microsphere imbedded in a cylindrical, slab, or a spherical shield). Accordingly, the calculations were performed using the concentric sphere option of the ISO-PC Code (IGEOM = 3).

Using the internal radiation-generating device photon results of Fig. 6 as a guide, candidate nuclides were limited to low-energy beta-gamma emitting isotopes. Table 4 summarizes the results of the ISO-PC calculations.

**Table 4:** Candidate low-energy beta-gamma emitting nuclides for loading microspheres.

Nuclide	Half-life	ISO-PC nuclide library number	Activity to deliver 100 Gy at 15 µm depth in an arteriole wall (kBq)
^32^P	14.28 d	459	81.7
^33^P	25.3 d	056	762
^35^S	87.2 d	460	440
^47^Sc	3.349 d	463	47.6
^72^Se	8.5 d	409	1.95
^82^Sr	25.36 d	540	1.40
^83m^Kr	1.86 h	045	1060
^90^Y	2.669 d	084	311
^99m^Tc	6.008 h	140	457
^103^Pd	16.99 d	570	3.64
^125m^Te	58.0 d	270	1.35
^125^I	59.4 d	595	1.09
^169^Er	9.39 d	630	1420
^189^Ir	13.2 d	665	0.475
^193m^Pt	14.33 d	677	384

The activity to deliver 100 Gy is obtained in a similar manner as the alpha microsphere calculation. ISO-PC calculates a dose rate $\dot {D}(0)$ for an initial activity *A*(0). The total dose (*D_T_*) is delivered by the complete decay of the activity *A*(0). To obtain the activity *A*′ that delivers 100 Gy to the arteriole wall, a relationship analogous to Eq. (7) is utilized:

(8) \begin{eqnarray*} \displaystyle {A}^{{\prime}}=A(0)\frac{100 \hspace{0.167em} \mathrm{Gy}}{{D}_{T}}=A(0)\frac{100 \hspace{0.167em} \mathrm{Gy}}{\dot {D}(0)}\lambda .&&\displaystyle \end{eqnarray*}

As defined in the alpha microsphere calculation, *t* = 0 corresponds to the time the microsphere attaches to the arteriole wall.

The half-life, ISO-PC nuclide library reference number, and activity to deliver 100 Gy to a depth of 15 µm into the arteriole wall are provided for each nuclide listed in Table 4. ISO-PC library reference numbers define the energy structure of source nuclides with specific details provided by Rittmann (2004). Calculations are also provided for ^32^P and ^90^Y to facilitate a comparison with currently utilized vascular disruption radionuclides that have been utilized in microsphere applications.

The ISO-PC model predicts that ^32^P and ^90^Y microspheres would require 81.7 and 311 kBq, respectively to deliver 100 Gy to the selected arteriole wall location. As noted in Eq. (8) the activity to deliver 100 Gy depends on the radiation type and energy of the radionuclide embedded in the carbon microsphere as well as its half-life. Therefore, nuclides with similar energies and half-lives (e.g., ^125m^Te and ^125^I) require similar activity levels to achieve 100 Gy. For ^125m^Te and ^125^I, the requisite activity is 1–2 kBq. As noted in the photon discussion, the fabricated microsphere activity is larger than *A*(0) and depends on the specific fabrication process, administration protocol, and characteristics of the selected radionuclide.

The predicted activity levels to achieve 100 Gy are in a range that can be readily incorporated into the microsphere, and there are numerous radionuclide options for incorporation into beta-gamma microspheres. In addition, the various nuclides listed in Table 4 offer considerable flexibility in developing a beta-gamma microsphere.

The choice of a beta-gamma radionuclide for microsphere clinical trials will depend on its availability and compatibility with the final microsphere design. However, the diversity of isotopes summarized in Table 4 suggests that a number of options are available for beta-gamma microspheres to provide improved dose localization in comparison with ^90^Y . However, the results of Fig. 6 illustrate the fact that some dose is delivered beyond the vessel wall. However, less healthy tissue is affected in comparison to ^90^Y microspheres. The beta-gamma microsphere design (e.g., material composition, radionuclide distribution, and coating material) can be selected to minimize the dose delivered beyond the vessel wall.

### Dose localization

The previous sections of this paper calculated the absorbed dose in a tumor’s vasculature for candidate nuclides and radiation types and their associated energies. A key issue for therapy applications is verification that the absorbed dose is delivered to the vasculature of tumors but not to healthy tissue. To address this important issue, the ranges of candidate radiation types and radionuclides considered in the previous discussion are summarized in Table 5.

**Table 5:** Dose localization for candidate radionuclides and radiation types.

Radionuclide or radiation delivery approach	Radiation type emitted	Range (µm)	*E* (MeV)
^90^Y	*β* ^−^	1.1 × 10^4^	2.281^a^
^32^P	*β* ^−^	7.9 × 10^3^	1.709^a^
^33^P	*β* ^−^	5.9 × 10^2^	0.249^a^
^35^S	*β* ^−^	3.2 × 10^2^	0.1674^a^
IRGD^b^	p	10–95	0.5–2.3
IRGD^b^	*α*	15–75	3.0–8.0
IRGD^b^	^12^C	5–90	10.0–50.0
IRGD^b^	^20^Ne	10–85	30.0–110.0
IRGD^b^	^40^Ca	10–75	100.0–300.0
IRGD^b^	*γ*	0–20^c^	0.015–0.050
IRGD^b^	*γ*	0–70^d^	0.015–0.050

**Notes.**

a Maximum beta energy.

b Internal radiation-generating device (IRGD).

c The dose decreases by a factor of about 10^3^ over the listed depths.

d The dose decreases by a factor of about 10^4^ over the listed depths.

The range *R*(*E*) of the ions (p, ^4^He, ^12^C, ^20^Ne, and ^40^Ca) and beta particles noted in Table 5 are calculated using standard approaches. Ion ranges are determined from the stopping power through the relationship (Bevelacqua, 2005):

(9) \begin{eqnarray*} \displaystyle R(E)=\int \nolimits \nolimits _{{E}_{i}}^{0}{\left(\frac{d E}{d x}\right)}^{-1}\hspace{0.167em} d E=\int \nolimits \nolimits _{{0}_{i}}^{{E}_{i}}{\left(-\frac{d E}{d x}\right)}^{-1}d E&&\displaystyle \end{eqnarray*}

where *E_i_* is the initial ion energy. The range of the beta particles is based on experimental results that have been fit to the standard relationship (Bevelacqua, 2010b):

(10) \begin{eqnarray*} \displaystyle R=412{E}_{m}^{1.265-0.0954\ln ({E}_{m})}&&\displaystyle \end{eqnarray*}

where *R* is the range in mg/cm^2^ and *E_m_* is the maximum beta energy in MeV. The physical range (*t*) is related to the density (*ρ*) of the medium attenuating the beta particle:

(11) \begin{eqnarray*} \displaystyle t=\frac{R}{\rho }.&&\displaystyle \end{eqnarray*}

Calculations of the photon range are based on the distance required to reduce the tumor vessel wall entrance photon dose by the factors noted in Table 5. Although this is somewhat arbitrary, it is sufficient to demonstrate that the dose delivered outside the blood vessel wall is at least a factor of 10^3^ smaller than the dose delivered to the surface of the blood vessel wall.

The results summarized in Table 5 illustrate that the candidate radionuclides and radiation types have a significantly shorter range than the ^90^Y or ^32^P beta particles. For example, a comparison of the following nuclides (beta ranges) illustrates the improvement in dose localization that can be achieved: ^90^Y (1.1 × 10^4^ µm), ^33^P (5.9 × 10^2^ µm) and ^35^S (3.2 × 10^2^ µm).

Beta emitting radionuclides have associated bremsstrahlung that irradiates tissue beyond the beta particle’s range. In comparison to ^90^Y , ^33^P and ^35^S bremsstrahlung have lower energies and a significantly shorter range. Consequently, the bremsstrahlung from ^33^P and ^35^S will irradiate less healthy tissue.

The alpha particles considered in Table 3 deposit their energy into <100 µm, which is <1% of the range of the ^90^Y beta particles. The shorter alpha particle range significantly limits the absorbed dose delivered to healthy tissue. Dose localization, with ranges <100 µm, is also achieved by ^12^C, ^20^Ne, and ^40^Ca ions. Low energy photons (15–50 keV) will have a shorter range than the ^90^Y bremsstrahlung, but will irradiate more healthy tissue than the low energy beta emitters (e.g., ^33^P and ^35^S). However, the candidate radionuclides and radiation types summarized in Table 5 demonstrate that improved absorbed dose localization for vascular disruption is possible and offer the potential for improvements when compared to ^90^Y .

## Candidate Cancer Types and Delivery Methods

This paper presents calculated doses for the selected radionuclides and radiation types. Although this is an interesting result, it is also of importance to describe the probable delivery methods of these radioactive materials to proposed target tumors. This is an important consideration to facilitate a more critical review of the feasibility of the proposed approach and to further its development.

A number of tumor types would be suitable candidates for the microsphere therapy modality that focuses on vascular disruption. In principle, any cancer type could be treated with microspheres intended to attach to the tumor’s vasculature. The limiting factor is ensuring that the microspheres preferentially attach to the tumor’s vasculature and do not deposit in locations that could irradiate healthy tissue.

In the initial approach, it is likely that the microspheres will be directed into the tumor vasculature using a catheter. The catheter will likely enter through an artery or vein to reach the candidate tumor vasculature. For example, a likely, initial cancer candidate would be liver tumors. Following the ^90^Y microsphere liver treatment delivery method, a catheter would be guided through the femoral artery into the liver and deliver the microspheres into the tumor’s vasculature. Image-guided radiation therapy (Lee & Seong, 2012; National Cancer Action Team, 2012) could be utilized to facilitate guiding the catheter to specifically target the tumor vasculature instead of normal vasculature.

A number of tumors could be treated using the catheter delivery approach. For example, access through the renal artery would facilitate microsphere delivery to tumors residing in the kidneys. Other cancer locations (artery or vein access) include: lungs (pulmonary artery), head and neck (carotid artery), and stomach, intestines and liver (hepatic portal system). Arteries and veins connect with appropriate blood vessel subsystems to provide a microsphere pathway to the desired locations. The specific catheter path for the various tumor types will be refined and developed in a manner that was similar to the evolution of the ^90^Y microsphere treatment of liver cancers (Carmeliet & Jain, 2000; Kennedy & Salem, 2003; Carr, 2004; Murthy et al., 2005; Kennedy et al., 2007; Chaudhury et al., 2010; Rajput & Agrawal, 2010)

The modality could be expanded to a more global set of tumors by incorporating nanotechnology into the construction of a second-generation microsphere. Appropriate nanotechnology would permit the spheres to be guided to the location of interest following injection into the body. Upon reaching the desired location the microspheres would be activated and attach to the wall of the tumor’s vasculature.

These two approaches require the prior detection of a tumor to initiate treatment. These tumors are well-established and are of sufficient size to permit detection. A significantly more advanced microsphere approach would involve the detection of tumors in their infancy and target the microspheres to the specific location of an infant tumor. This approach would utilize a smaller quantity of radioactive material since the tumors would be small and their vasculature structures more susceptible to attack.

## Prospective and Significance

The proposed vascular disruption approach is currently a theoretical endeavor. Ideally, it would have an experimental basis, but that is beyond the scope of this paper. Future experimental effort will be based on the calculations of this paper as well as development of the detailed microsphere design. For example, this paper presupposes that the microspheres will preferentially attach to a tumor’s blood vessels and avoid the issues associated with transporting radioactive material to other tissues.

Accomplishing preferential attachment requires significant development. For example, the microsphere design effort will evaluate a variety of physical approaches (e.g., electric charge, heat, pH, and electromagnetic fields) to facilitate the preferential attachment of the microsphere to the tumor’s vascular wall. The specific microsphere design characteristics that require experimental investigation include its material composition, electric charge and its spatial distribution, physical size and shape, and dielectric and diamagnetic characteristics. The final microsphere design may be enhanced through the use of activating agents which could include the use of lasers, heat, electric fields, magnetic fields, microwaves, and a spectrum of radiofrequency radiation. Both the microsphere design and activating agents require extensive verification efforts. These investigations and the results of this paper are required before a microsphere experiment can be performed. Accordingly, no experimental data associated with tumor vascular disruption are included in this paper.

## Conclusions

^32^P and ^90^Y has been incorporated into microspheres to facilitate the radioembolization of a tumor’s vascular structure. Although these high-energy beta emitters effectively irradiate the vessel wall, their range and associated bremsstrahlung also irradiate healthy tissue lying outside the vasculature.

Internal radiation-generating devices have the potential to localize dose in the tumor vasculature and facilitate its disruption. Although beyond current technology, these devices suggest a variety of radiation types including protons, alpha particles, ^12^C ions, ^20^Ne ions, ^40^Ca ions, and low-energy photons can be used to facilitate vascular disruption. The internal radiation generating device absorbed dose calculations suggest that low-energy beta-gamma and alpha emitting radionuclides incorporated into a microsphere are additional options to localize dose in a tumor’s vasculature.

Specific microsphere calculations utilizing beta-gamma and alpha emitting radionuclides suggest a number of nuclides are candidates to replace ^90^Y in microsphere applications. Each of these nuclides provides improved dose localization and minimizes the dose beyond the target tumor vasculature. However, considerable development is required to design an appropriate microsphere loaded with alpha or low-energy beta-gamma emitting radionuclides and to verify its capability to perform as designed. If appropriately designed, these various beta-gamma and alpha microspheres have significant potential to minimize the absorbed dose delivered to non-target tissues.
